# Downregulation of PAQR3 expression predicts poor prognosis of patients with pan-cancer: a meta-analysis

**DOI:** 10.3389/fonc.2025.1574150

**Published:** 2025-05-05

**Authors:** Kui Zhai, Xing-Peng Miao, Shi-Gui Ai, Qiang Guo, Ren-Zhong Zha

**Affiliations:** ^1^ Department of Thoracic Surgery, Xingyi People’s Hospital, Xingyi, Guizhou, China; ^2^ Department of Cardiothoracic Surgery, Taihe Hospital, Taihe Hospital, Hubei University of Medical, Shiyan, China

**Keywords:** PAQR3, cancer, meta-analysis, biomarker, EGFR

## Abstract

This study aimed to evaluate the association between progestin and adipoQ receptor 3 (PAQR3) expression and survival outcomes, as well as disease progression, through a meta-analysis. A systematic literature search was conducted across multiple academic databases, including Cochrane Library, Embase, PubMed, Web of Science, and Google Scholar. Relevant studies were identified and screened based on predefined inclusion and exclusion criteria. Statistical analyses, including odds ratios (ORs), hazard ratios (HRs), and 95% confidence intervals (CIs), were employed to assess the relationship between PAQR3 expression levels and prognostic outcomes, as well as clinicopathological features in pan-cancer patients. A total of 1032 patients from nine eligible studies were included in the meta-analysis. The results demonstrated that high PAQR3 expression was significantly associated with improved overall survival (HR = 0.32; 95% CI = 0.19–0.56) and disease-free survival (HR = 0.35; 95% CI = 0.20–0.61) in pan-cancer patients. Downregulation of PAQR3 expression was significantly correlated with adverse clinicopathological characteristics, including lymph node metastasis (HR = 0.22; 95% CI = 0.06–0.82), higher histological grade (HR = 0.31; 95% CI = 0.12–0.81), advanced pathological stage (HR = 0.18; 95% CI = 0.05–0.66), tumor size (HR = 0.73; 95% CI = 0.36–1.5), distant metastasis (HR = 0.36; 95% CI = 0.07–1.82), and tumor invasion (HR = 0.18; 95% CI = 0.11–0.29). However, no significant associations were observed between PAQR3 expression and gender (HR = 0.94; 95% CI = 0.59–1.5) or age (HR = 1.10; 95% CI = 0.77–1.56). In conclusion, reduced PAQR3 expression in pan-cancer patients is associated with worse clinical outcomes and may promote cancer progression. These findings suggest that PAQR3 could serve as a potential prognostic biomarker and therapeutic target for cancer treatment.

## Introduction

Cancer is currently one of the major public health concerns worldwide. Early surgical intervention may improve survival rates in patients with cancer. However, some patients depict poor prognosis despite therapeutic modalities, such as surgery, radiotherapy, chemotherapy, or endocrine therapy. Recently, there has been an innovative development of some new molecular targeted drugs. Targeted therapy is known to be a promising treatment scheme for patients with poor prognosis (1–3). For example, epidermal growth factor receptor tyrosine kinase inhibitors (EGFR TKIs) targeted therapy can remarkably improve the prognosis of patients with EGFR-mutant advanced non-small cell lung cancer (NSCLC) and is considered as standard first-line treatment for these patients (2). However, there are still constraints in effectively treating lung adenocarcinoma by targeting EGFR mutations, as some patients with this condition may develop drug resistance, which can result in a poor prognosis (4). Thus, it is imperative to identify new targets for cancer treatment for patients with poor prognosis.

Progestin and adipoQ receptor 3 (PAQR3) is a transmembrane protein localized in the Golgi apparatus of mammalian cells and is a member of the PAQR family. Accumulating evidence has demonstrated that PAQR3 plays a critical role in suppressing extracellular signal-regulated kinase (ERK) signaling pathways in cancer (5–21). For instance, PAQR3 expression has been found to be significantly downregulated in various malignancies, including laryngeal squamous cell carcinoma (LSCC) and non-small cell lung cancer (NSCLC) (9–13). Notably, overexpression of PAQR3 has been shown to modulate ERK phosphorylation, thereby inhibiting cancer cell proliferation and invasion in LSCC (9). PAQR3 expression also affects cancer occurrence and development through several mechanisms, like PI3K/AKT, epithelial-mesenchymal transition (EMT), and NF-κB/p53/Bax (10–13). Additional studies report that PAQR3 could inhibit the growth and proliferation of several cancers including esophageal cancer, gastric cancer, prostate cancer, and other cancer (8–10, 12, 15, 18–21). However, the clinical importance of PAQR3 in the prognosis of tumor patients has not yet been completely analyzed via meta-analysis. Therefore, this study explores the roles of PAQR3 in prognosis and clinicopathological features of cancer patients through a comprehensive meta-analysis. We aim to develop a new theoretical framework for the treatment of patients with cancer.

## Methods

### Retrieval of studies

On January 1, 2024, a systematic search of both domestic and international research was planned. To retrieve eligible studies, a literature search was performed in the following databases: Cochrane Library, Embase, PubMed, Web of Science, CNKI, Wanfang database and Google Scholar. The search, aimed to examine the correlation between PAQR3 expression and prognosis and clinical pathological characteristics of cancer patients, was performed using the keywords “Progestin and adipoQ receptor 3” or “PAQR3” and “Tumor” or “Cancer”.

### Inclusion and exclusion criteria

The inclusion criteria for eligible studies were as follows: 1) human cancer patients included in the study; 2) real-time quantitative polymerase chain reaction (PCR) or reverse transcription PCR was used to determine PAQR3 expression in cancer tissues; 3) a clear association between PAQR3 expression and disease-free survival (DFS), clinical pathological features, or overall survival (OS) of cancer patients was established; 4) categorization of patients as high- or low- PAQR3 groups based on the PAQR3 levels; 5) information on 95% confidence interval (CI), hazard ratio (HR), and odds ratio (OR) provided in the study; 6) studies published in English language. The exclusion criteria for the articles included: 1) study on animal subjects; 2) incomplete data collection; 3) insufficient data on clinical research of cancer patients.

### Data extraction and quality evaluation

**Figure 1:**
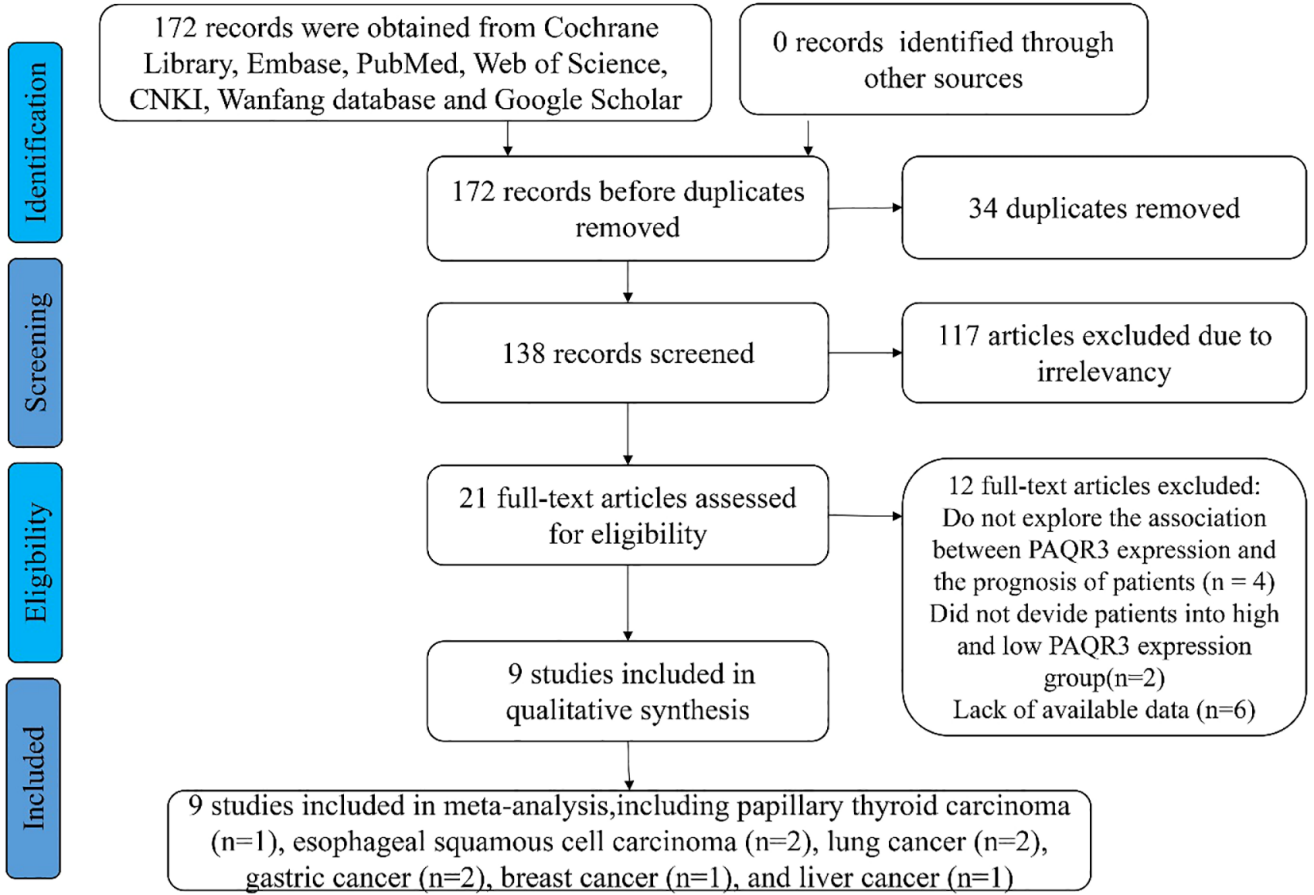
The flow diagram of eligible studies in this meta-analysis.

The data was extracted independently by two researchers based on established screening criteria and evaluated by a third professional in case of disagreement (**Figure 1**). This meta-analysis is currently undergoing the registration process. Therefore, the registration number and name of the meta-analysis cannot be disclosed at this time. The extracted data contains the following information: 1) publication year, country of research, and first author; 2) patient characteristics, including cancer type, total number of patients from each study, and number of subtype patients; 3) empirical research result indicators such as lymph node metastasis, OS, pathologic stage, distant metastasis. The HR and 95% CI values were obtained using EngaugeDigitizeServerI10.1 (http://Digitizer.sourceforge.net/) software, and the HR and 95% CI values were matched with the P-value from the survival curve of the original articles. The quality of articles was assessed via the Newcastle-Ottawa quality assessment scale method based on group selection, comparability, and exposure or outcome evaluation. Moreover, this study uses a semi-quantitative approach utilizing a star system in conjunction with the Newcastle-Ottawa quality assessment scale to assess the quality of the articles (22). A perfect score of 9 stars was defined, whereas the articles with more than 5 stars were considered high-quality research reports. Additional, tumor size and invasion depth were categorized based on the criteria provided in the original literature. For instance, if the original literature used 3 centimeters as a cutoff, then tumor diameters were divided into large and small categories.

### Statistical analysis

The statistical analysis was conducted using STATA 12.0 (Stata Corp, College Station, Texas, USA) and RevMan 5.4.0 software (Cochrane Collaboration, London, UK). The study endpoint was evaluated using HR and 95% CI values while clinical pathological characteristics of patients were measured using OR and 95% CI values. The heterogeneity of research results was quantitatively evaluated using I^2^ and P-values. When P ≥ 0.05 or I^2^ ≤ 50%, a fixed effects model of Mantel-Haenszel was utilized. In contrast, when P < 0.05 or I^2^ > 50%, a random effects model was employed due to significant heterogeneity in the results. The presence of publication bias was evaluated using the Begg test method. Meanwhile, the stability of the research was assessed through a sensitivity analysis. In addition, P < 0.05 was considered in this study.

## Results

### Study characteristics

A total of 172 relevant articles were obtained based on the search strategy. Eventually, 34 articles were excluded due to duplication, and 117 articles were foreclosed after reading titles and abstracts. After a detailed reading of the full text, 12 publications were found to be inconsistent with the inclusion criteria. Of the 12 studies, 4 articles showed PAQR3 and OS or DFS of patients were not related, two articles lacked PAQR3 grouping criteria, and 6 articles included incomplete data (**Figure 1**). A total of nine studies involving 1032 patients were finally included in the present meta-analysis (**Table 1**). These nine studies focused on different cancer types, including papillary thyroid carcinoma (14), esophageal squamous cell carcinoma (ESCC) (8, 15), lung cancer (16, 17), gastric cancer (18, 19), breast cancer (20), and liver cancer (21). All included studies were conducted in China and met the standards of high-quality articles (**Table 2**).

**Table 1:** The basic characteristics of the included literature.

Author	Cancer	Expression	Reference gene	N	Cut-off value	Outcome	HR value with 95%CI	Follow-up month	NOS score
Gao et al. (14)	PTC	Down-regulated	not reported	60	Not reported	NA	Not reported	NA	6
Bai et al. (8)	ESCC	Down-regulated	β-actin	40	Median	NA	Not reported	NA	6
Shen et al. (16)	LUAD	Down-regulated	not reported	86	Not reported	OS	0.189 (0.072-0.923)	60	8
Wu et al. (18)	GC	Down-regulated	β-actin	146	Not reported	NA	Not reported	NA	6
Bai et al. (15)	ESCC	Down-regulated	β-actin	80	Median	OS	0.275 (0.077-0.980)	60	9
						DFS	0.568 (0.337-0.952)		
Li et al. (20)	BC	Down-regulated	β-actin	82	Median	OS	0.136 (0.0527-0.352)	60	9
						DFS	0.36 (0.098-1.315)		
Wu et al. (21)	HCC	Down-regulated	GAPDH	132	Median	OS	0.608 (0.375-0.984)	100	9
						DFS	0.425 (0.307-0.722)		
Ling et al. (19)	GC	Down-regulated	GAPDH	300	Mean	OS	0.1779 (0.0298-1.064)	60	9
						DFS	0.149 (0.0768-0.289)		
Liang et al. (17)	LC	Down-regulated	β-actin	106	Not reported	OS	0.56 (0.33-0.95)	60	8

LC, lung cancer; NOS, Newcastle-Ottawa Scale; PTC, papillary thyroid carcinoma; GC, gastric cancer; BC, breast cancer; HCC, hepatocellular carcinoma; DFS, disease-free survival; GAPDH, Glyceraldehyde-3-phosphate dehydrogenase; ESCC, esophageal squamous cell carcinoma; NA, non-available; OS, overall survival.

**Table 2:** Quality assessment of eligible studies Newcastle-Ottawa scale score.

Author and year	Country	Selection				Comparability	Outcome			Total
		Adequate of case definition	Representativeness of the cases	Selection of Controls	Definition of Controls	Comparability of cases and controls	Ascertainment of exposure	Same method of ascertainment	Non-Response rate	
Gao et al. (14)	China	*	*	*	*	*	–	*	–	6
Bai et al. (8)	China	*	*	*	*	*	–	*	–	6
Shen et al. (16)	China	*	*	*	*	*	*	*	*	8
Wu et al. (18)	China	*	*	*	*	*	–	*	–	6
Bai et al. (15)	China	*	*	*	*	**	*	*	*	9
Li et al. (20)	China	*	*	*	*	**	*	*	*	9
Wu et al. (21)	China	*	*	*	*	**	*	*	*	9
Ling et al. (19)	China	*	*	*	*	**	*	*	*	9
Liang et al. (17)	China	*	*	*	*	*	*	*	*	8

*, The above content is described in the original article; **, The above content is clearly described in the original article.

### Overexpression of PAQR3 significantly related to OS and DFS of pan-cancer patients

**Figure 2:**
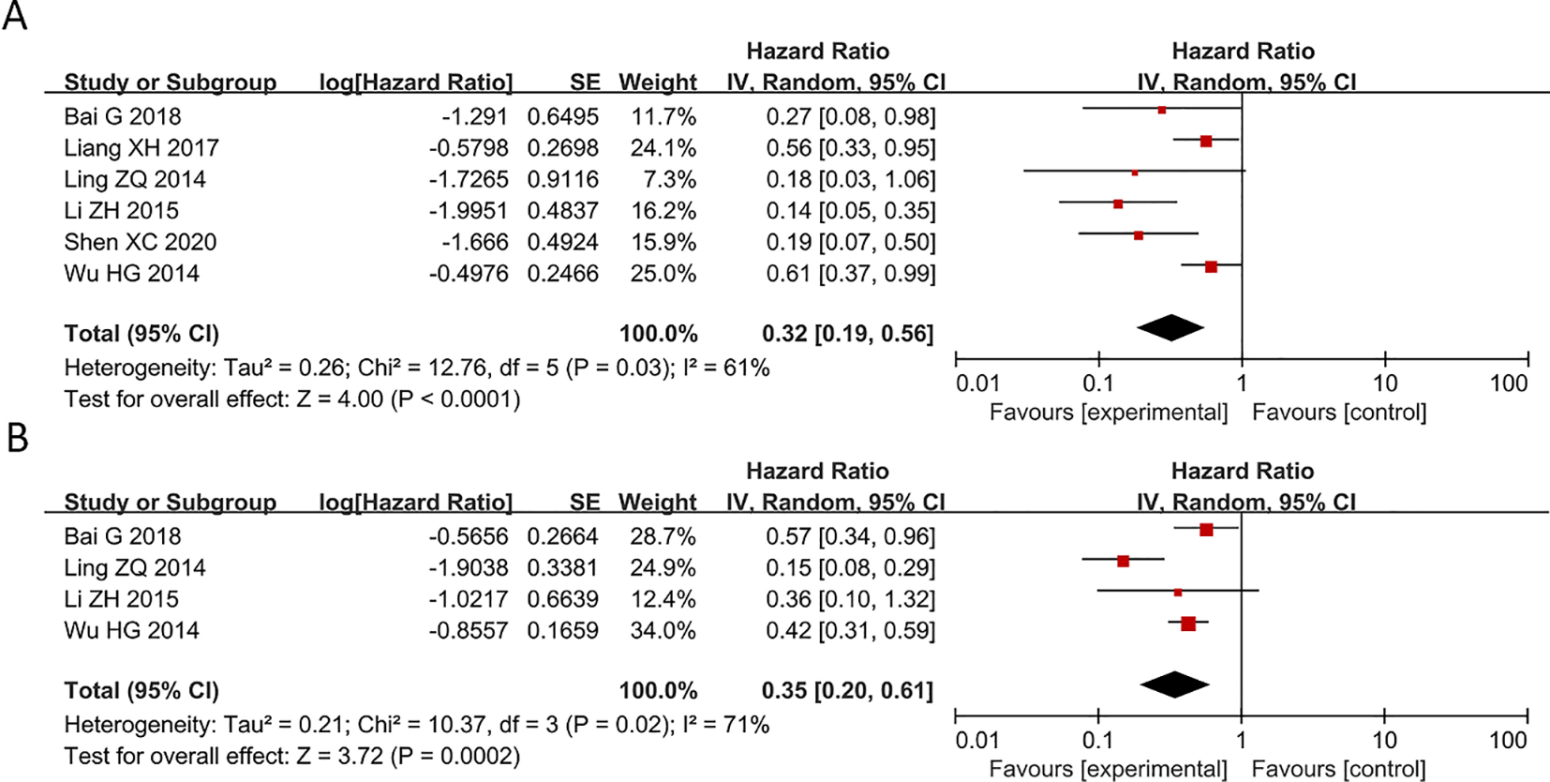
Forest plot of HRs for the association between PAQR3 expression and cancer prognosis stratified by different cancer types. **(A)** OS, **(B)** DFS. DFS, disease-free survival; OS, overall survival.

In the included studies, 6 publications discussed OS and DFS of pan-cancer patients (**Figure 2**). All these six articles explored the relationship between PAQR3 levels and the OS and four studies investigated the association between PAQR3 levels and DFS of cancer patients. Specifically, after merging HR with 95% CI, a decrease in PAQR3 expression levels was significantly related to poor OS in 786 patients (HR = 0.32, 95% CI:0.19-0.56; **Figure 2A**). Due to significant heterogeneity (I^2^ = 61%; P<0.05), a random effects model was applied to this meta-analysis. Decreased PAQR3 levels were also significantly related to poor DFS in 594 cancer patients (HR=0.35, 95% CI: 0.20-0.61; **Figure 2B**). Due to significant heterogeneity (I^2^ = 71%; P<0.05), a random effects model was applied to this meta-analysis. These results indicate better survival time in cancer patients with PAQR3 overexpression.

### Decreased expression of PAQR3 significantly related to pathological stage, lymph node, and distant metastasis

**Figure 3:**
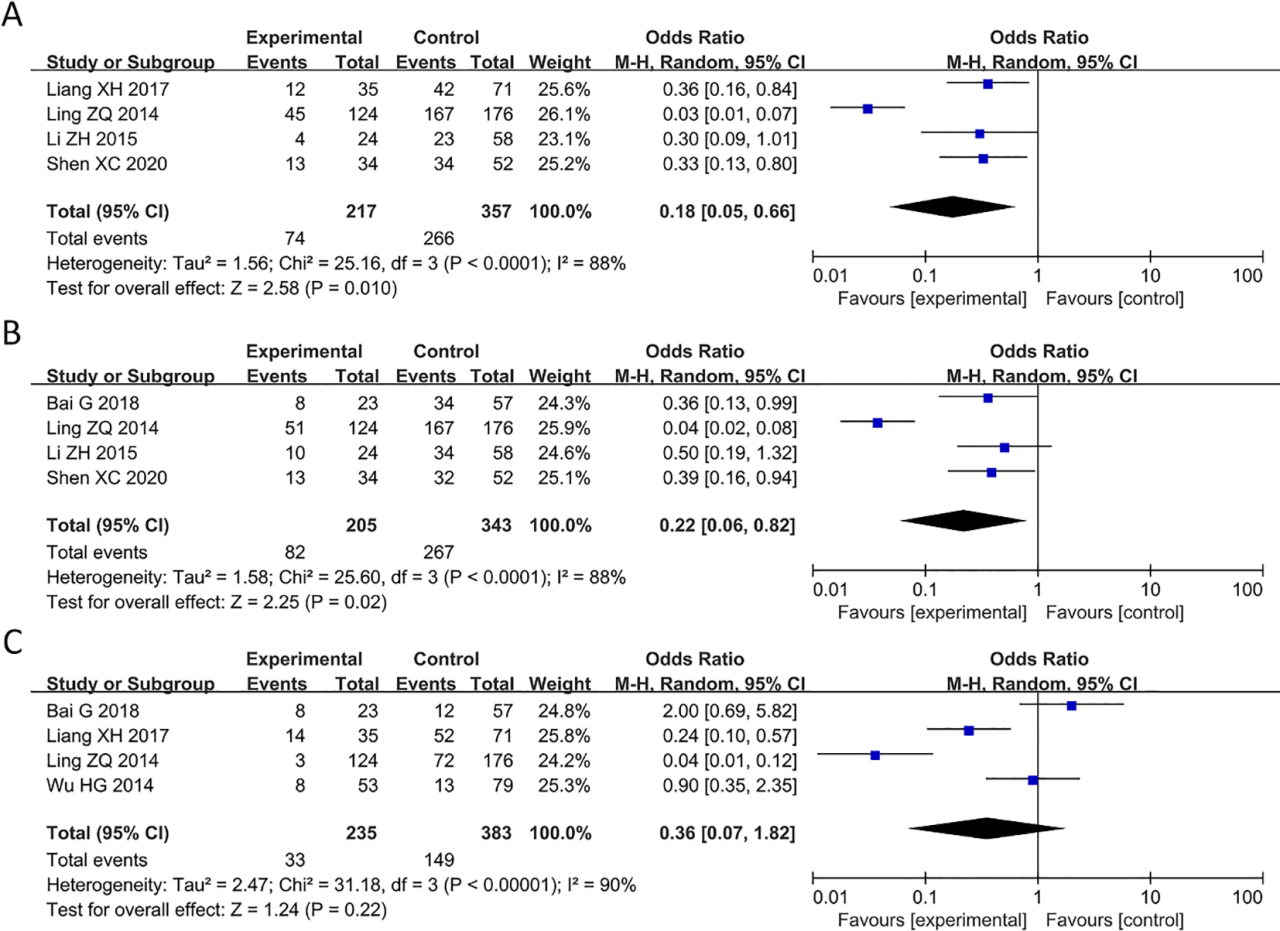
Forest plot of HRs for the association between PAQR3 expression and clinical pathological stage, lymph node metastasis, and distant metastasis stratified by different cancer types. **(A)** Pathological stage, **(B)** Lymph node metastasis, **(C)** Distant metastasis.

Of the 9 articles analyzed, 4 studies reported a correlation between PAQR3 levels and TNM stage (pathological stage) of cancer patients (**Figure 3A**). A random effect model was applied for obvious heterogeneity (I^2^ = 88%; P<0.05). The HR and 95% CI for patients with pathological stage were 0.18 and 0.05-0.66, respectively, indicating that patients with overexpression of PAQR3 tend to have earlier pathological stage as compared to patients with low PAQR3 expression levels (**Figure 3A**). Additionally, 4 articles reported a significant correlation between PAQR3 levels and lymph node metastasis in patients (HR = 0.22, 95%CI: 0.06-0.82; **Figure 3B**). The heterogeneity of the results was significant (I^2^ = 88%; P<0.05), and a random effects model was applied. These results indicate patients with decreased PAQR3 expression levels tend to have lymph node metastasis compared to those with PAQR3 overexpression (**Figure 3B**). Lastly, 4 articles reported a significant correlation between decreased PAQR3 levels and distant metastasis in cancer patients (HR= 0.36, 95%CI: 0.07-1.82; **Figure 3C**). The heterogeneity of the results was significant (I^2^ = 90%; P<0.05), and a random effects model was applied in this meta-analysis.

### Correlation between decreased expression of PAQR3 and tumor size, histological grade, and invasion depth

**Figure 4:**
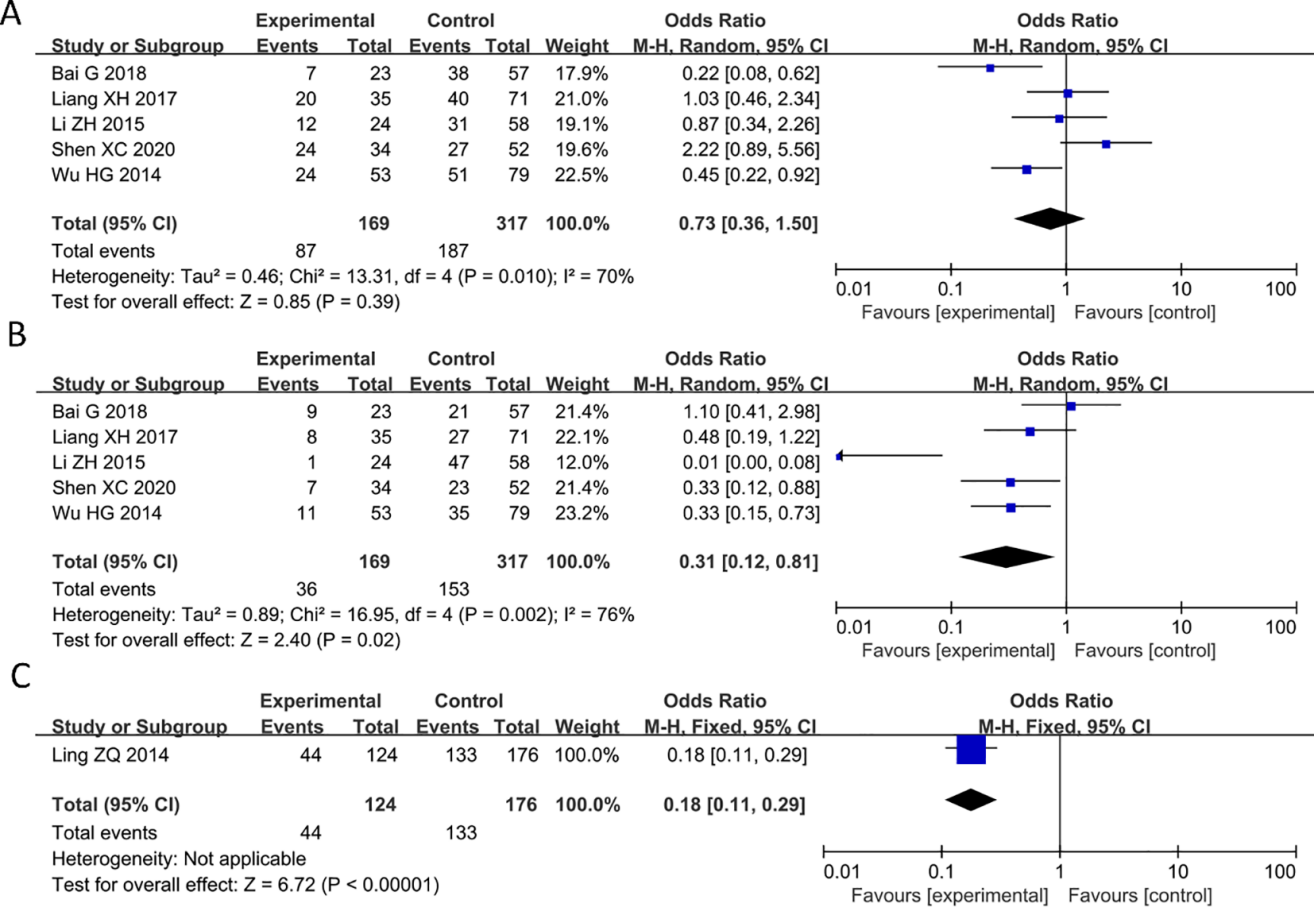
Forest plot of HRs for the association between PAQR3 expression and tumor size, histological grade, and invasion depth stratified by different cancer types. **(A)** Tumor size, **(B)** Histological grade, **(C)** Depth of invasion.

Of the 9 articles analyzed, 5 studies reported a significant correlation between decreased expression of PAQR3 and tumor size in cancer patients (HR = 0.73, 95%CI: 0.36-1.5; **Figure 4A**). The heterogeneity of the results was significant (I^2^ = 70%; P< 0.05), and thus a random effects model was applied in this meta-analysis. Additionally, 5 studies reported a significant correlation between decreased expression of PAQR3 and histological grade in cancer patients (HR = 0.31, 95%CI: 0.12-0.81; **Figure 4B**). The heterogeneity of the results was significant (I^2^ = 76%; P<0.01), and thus a random effects model was applied in this meta-analysis. Lastly, 1 study reported a significant correlation between the expression level of PAQR3 and invasion depth in cancer patients (HR = 0.18, 95%CI: 0.11-0.29; **Figure 4C**). These results suggest that low expression levels of PAQR3 imply the presence of larger tumor diameter, low histological grade, and higher invasion depth.

### No significant correlation between decreased PAQR3 levels and age and gender

**Figure 5:**
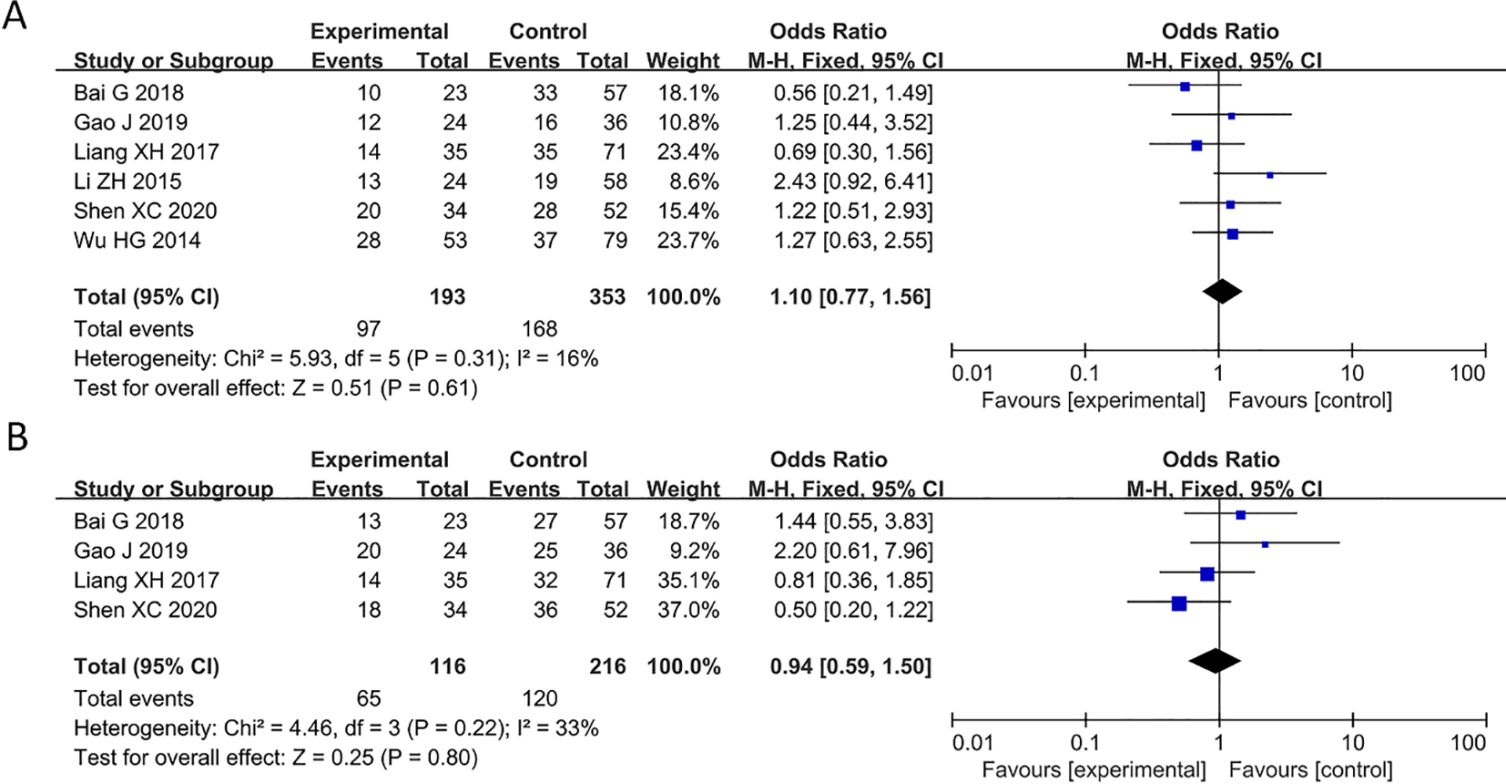
Forest plot of HRs for the association between PAQR3 expression and age and gender stratified by different cancer types. **(A)** Age, **(B)** Gender.

6 studies reported relationship between decreased PAQR3 level and age of cancer patients (HR = 1.1, 95%CI: 0.77-1.56; **Figure 5A**). The heterogeneity of the results was not significant (I^2^ = 16%; P>0.05), and thus a fixed effects model was applied in this meta-analysis. Additionally, 4 articles reported an association between decreased PAQR3 expression and gender of cancer patients (HR = 0.94, 95%CI: 0.59-1.5; **Figure 5B**). The heterogeneity of the results was not significant (I^2^ = 33%; P>0.05), and thus a fixed effects model was applied in this meta-analysis. These results indicate that there was no significant correlation between decreased PAQR3 levels and the age and gender of patients.

### Bias and sensitivity analysis

**Figure 6:**
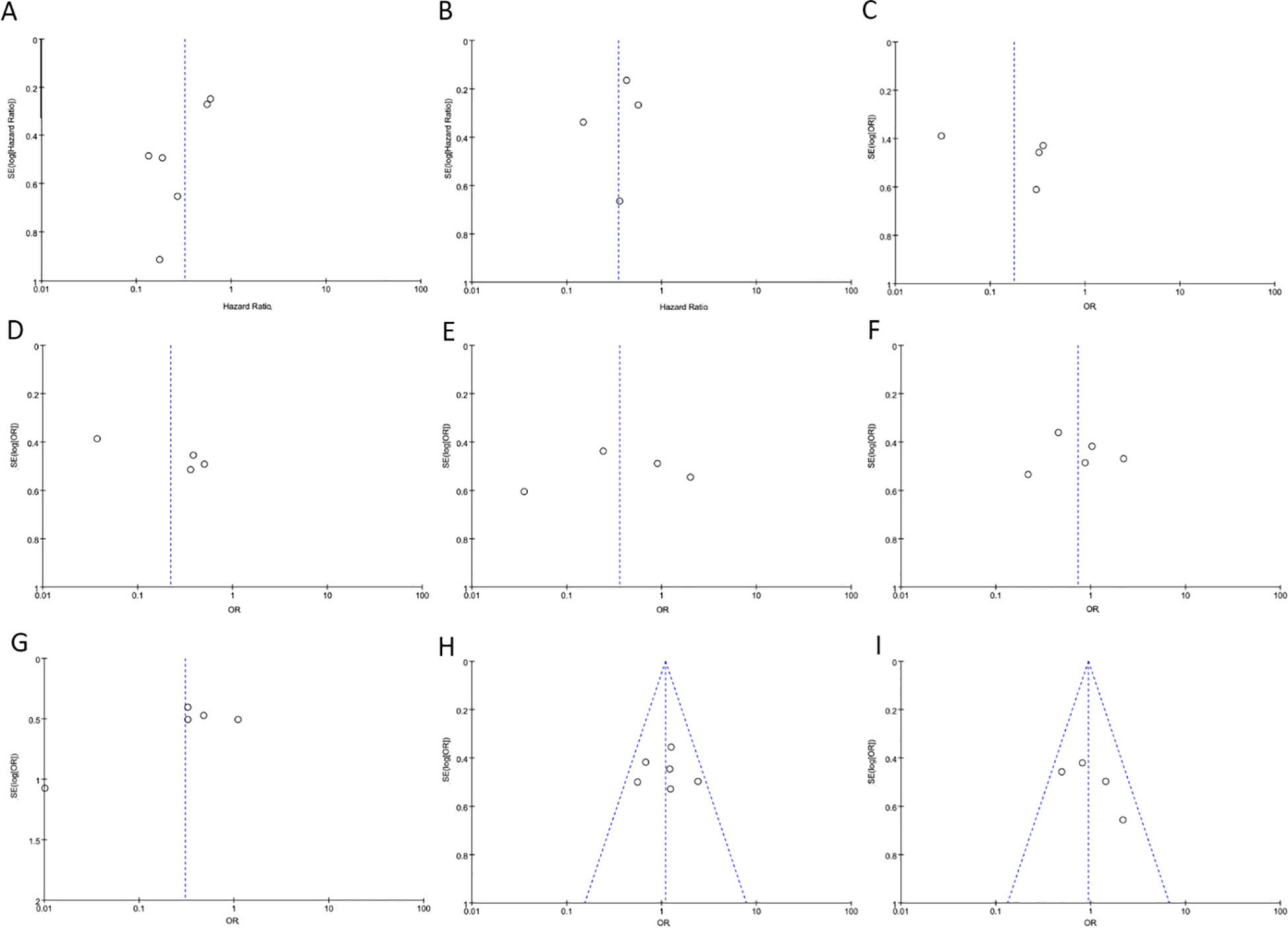
The funnel plot between PAQR3 expression and the clinicopathological parameters in various cancers. **(A)** OS; **(B)** DFS; **(C)** Pathological stage; **(D)** Lymph node metastasis, **(E)** Distant metastasis; **(F)** Tumor size; **(G)** Histological grade; **(H)** Age; **(I)** Gender. DFS, Disease-free survival; OS, overall survival.

**Figure 7:**
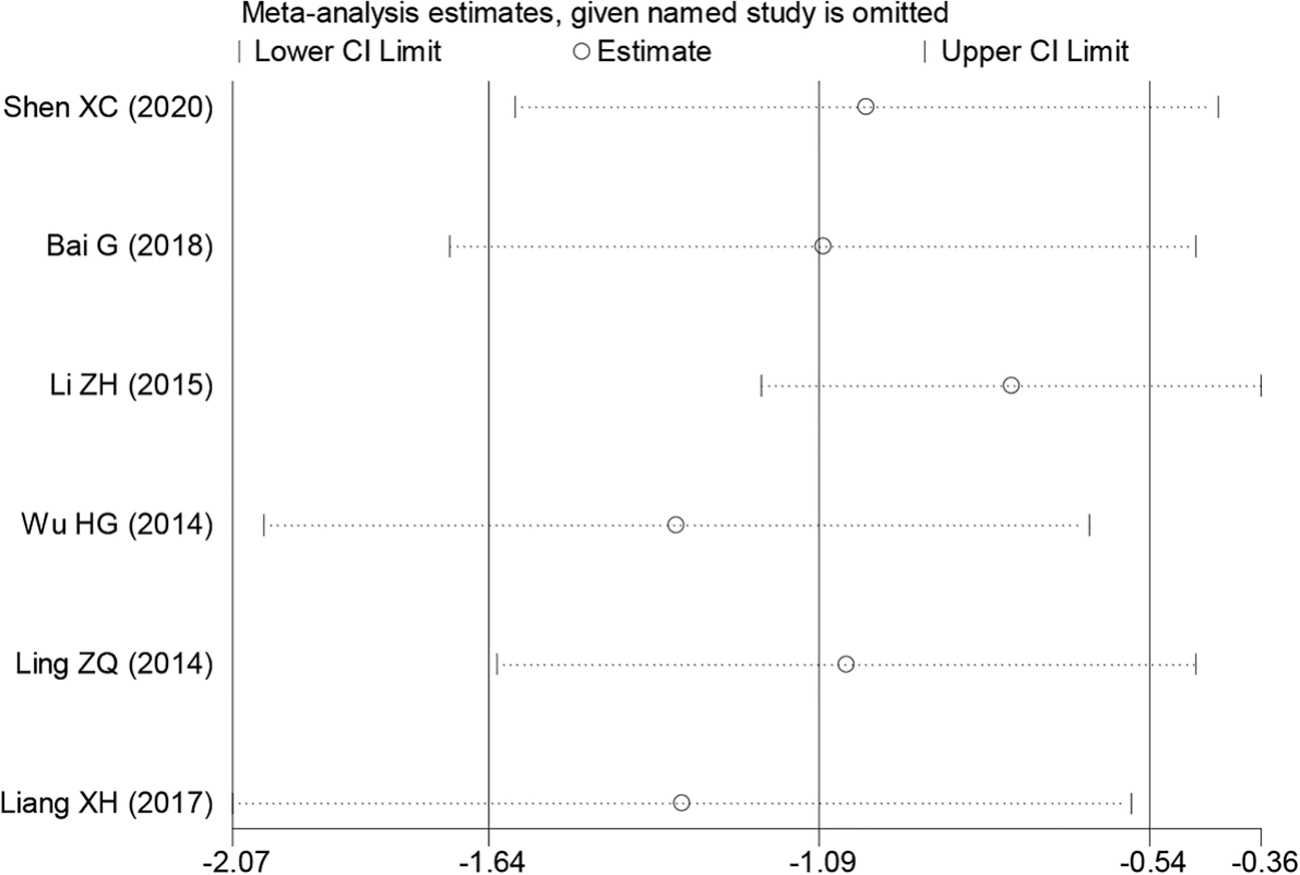
Sensitivity analysis for PAQR3 expression in detecting various cancer patients’ overall survival (OS). HR, hazard ratio; CI, confidence interval.

During the sensitivity analysis of prognostic indicators and clinical pathological features of cancer patients using Stata 12.0 software, the overall results did not show significant changes after removing individual studies, indicating that these studies did not have a significant impact on the overall results. In other words, each study included in the analysis was consistent with the overall results (**Figure 6**). Additionally, a funnel plot using Revman 5.4.0 showed no significant publication bias or other biases among the included studies (**Figure 6**). Therefore, we believe the results of this meta-analysis study to be trustworthy (**Figure 7**).

## Discussion

The rapid advancement of high-throughput gene sequencing technologies and The Cancer Genome Atlas (TCGA) database has revolutionized our understanding of the genetic mechanisms underlying human diseases, including cancer (23–28). During cancer progression, the expression of numerous genes becomes dysregulated, functioning either as oncogenes or tumor suppressors, and playing critical roles in tumor initiation and development (25–29). For instance, kynurenine 3-monooxygenase (KMO) is significantly downregulated in hepatocellular carcinoma tissues, correlating with poor prognosis in patients with hepatocellular carcinoma. Experimental studies have demonstrated that reduced KMO expression promotes proliferation, invasion, and metastasis while inhibiting apoptosis in liver cancer cells, partly through its involvement in the epithelial-mesenchymal transition (EMT) process (28). These results suggest that increased expression of KMO may become a potential therapeutic target for human cancer. Recently, the roles of PAQR3 in several cancers has been extensively studied, and decrease in PAQR3 expression is significantly associated with poor prognosis and progression in cancer patients (5–21). For example, PAQR3 expression is reported to be significantly lower in ESCC tissues than in adjacent normal tissues. Decreased expression of PAQR3 was found to be related to advanced clinical stage and lymph node positivity in patients with ESCC. Moreover, PAQR3 upregulation could inhibit colony formation, proliferation, and invasion of ESCC cells, and block cell cycle transition which could be the outcomes related to increased p27 and p21 expression and decreased expression of cyclin D1, CDK4, and CDK2 proteins (6). PAQR3 overexpression was also shown to disrupt the growth of ESCC cells through inhibition of ERK1/2 phosphorylation (8). In addition, Levels of PAQR3 were considerably reduced in NSCLC tissues and were associated with histological subtype, lymph node metastasis, and diagnosis of NSCLC in patients. Increased expression of PAQR3 was found to improve the OS of patients with lung adenocarcinoma. PAQR3 expression can lead to reduced cancer cell proliferation via NF-κB/P53/Bax signaling pathway and reducing the expression of PAQR3 can reverse its effects (13).

Meta-analysis could be utilized to determine the relationship between the expression of a specific protein and the prognosis in patients with different types of cancer through data that is already published. The roles of PAQR3 expression in the prognosis of patients with different types of cancer has not been fully analyzed. In this study, we analyzed 1032 patients with pan-cancer included in 9 different studies using meta-analysis (14–21) and found that PAQR3 overexpression was significantly related to improved OS and DFS of these patients. These results indicate that patients with elevated PAQR3 expression levels have longer survival time. In addition, the downregulation of PAQR3 is related to earlier pathological stage, lymph node metastasis, distant metastasis, larger tumor size, histological grade, and invasion depth in these patients. These results suggest that PAQR3 may become a biomarker for the prognosis of cancer patients.

In the present study, meta-analysis results implied that high expression of PAQR3 can delay cancer progression and improve cancer prognosis. In other words, enhancing PAQR3 expression is anticipated to improve outcomes for cancer patients. However, there are still some limitations in this study. Firstly, all the studies included in the meta-analysis are from China and the conclusion from this study may apply only to the Asian or Chinese population. Further collection of data from the European or South American population is required to identify the role and clinical importance of PAQR3 in cancer. Secondly, randomized controlled studies included in this meta-analysis have relatively small sample sizes that may lead to systematic bias. Large-scale studies are, therefore, required to support our results. Additionally, there may be extraction or calculation bias when extracting HR values from the Kaplan-Meier survival curve. Overall, this meta-analysis confirms downregulation of PAQR3 expression is related to poor prognosis in patients with pan-cancer. This will encourage researchers to further investigate the clinical roles and molecular regulatory mechanisms associated with PAQR3.

## Conclusion

The decreased expression of PAQR3 in pan-cancer might promote cancer progression and PAQR3 may be a potential therapeutic target and prognostic biomarker for cancer patients.
